# MiR-3188 Inhibits Non-small Cell Lung Cancer Cell Proliferation Through FOXO1-Mediated mTOR-p-PI3K/AKT-c-JUN Signaling Pathway

**DOI:** 10.3389/fphar.2018.01362

**Published:** 2018-12-11

**Authors:** Chunyan Wang, Enqi Liu, Wen Li, Jue Cui, Tongxiang Li

**Affiliations:** College of Food and Biology Engineering, Xuzhou Institute of Technology, Xuzhou, China

**Keywords:** miR-3188, NSCLC, proliferation, mTOR, PI3K/AKT, c-JUN

## Abstract

This study investigated the role of miR-3188 on the proliferation of non-small cell lung cancer cells and its relationship to FOXO1-modulated feedback loop. Two non-small cell lung cancer (NSCLC) cell lines A549 and H1299 were used. RNA silencing was achieved by lentiviral transfection. Cell proliferation was assessed by immunohistochemical staining of Ki67 and PCNA, Edu incorporation, and colony formation assay. Western blotting was used to examine expression of FOXO1, mTOR, p-mTOR, CCND1, p21, c-JUN, AKT, pAKT, PI3K, p-PI3K, and p27 proteins. It was found that miR-3188 reduced cell proliferation in NSCLC cells. Molecular analyses indicated that the effect of mammalian target of rapamycin (mTOR) was directly mediated by miR-3188, leading to p-PI3K/p-AKT/c-JUN inactivation. The inhibition of this signaling pathway further caused cell-cycle suppression. Moreover, FOXO1 was found to be involved in regulating the interaction of miR-3188 and mTOR through p-PI3K/p-AKT/c-JUN signaling pathway. Taken together, our study demonstrated that miR-3188 interacts with mTOR and FOXO1 to inhibit NSCLC cell proliferation through a mTOR-p-PI3K/AKT-c-JUN signaling pathway. Therefore, miR-3188 might be a potential target for the treatment of NSCLC.

## Introduction

MicroRNAs (miRNAs or miRs) play important roles in development, cellular differentiation, proliferation, cell cycle control, and cell death (Bhaskaran and Mohan, 2014). They are critical in development and progression of various kinds of diseases including cancer (Hayes et al., 2014; Rupaimoole and Slack, 2017). Since it was found that the miRNA was involved in chronic lymphocytic leukemia, more and more miRNAs were identified to link with development and progression of cancers (Musilova and Mraz, 2015). It has been reported that miRNAs can regulate chemotherapeutic efficacy in multiple cancers (Magee et al., 2015; Mognato and Celotti, 2015). Hui-Ming Lin et al have discovered that miR-217 and miR-181b-5p significantly enhanced apoptosis in PC3 cells, indicating their therapeutic potential to improve taxane response in castration-resistant prostate cancer (CRPC) (Lin et al., 2018). Moreover, Bone marrow-derived mesenchymal stem/stromal cells (BM-MSCs)-derived exosomes promote colon cancer stem cell-like traits via miR-142-3p, suggesting multiple ways of miRNAs in regulatiing cancer progression (Li and Li, 2018). As one of the original miRNAs discovered, the biological role of miR-3188 and its molecular mechanisms underlying cancer initiation and progression has been reported in nasopharyngeal carcinoma (Zhao et al., 2016). Additionally, miR-3188 suppression could inhibit proliferation of breast cancer cells through regulating p27 expression. However, how it works in non-small cell lung cancer remains unclear.

Forkhead box protein O1 (FOXO1) is a protein encoded by the FOXO1 gene. FOXO1 shuttles between nucleus and cytoplasm back and forth. AKT phosphorylates FOXO1 and leads to FOXO1 translocate from nucleus to cytoplasm (Hay, 2011; Tzivion et al., 2011). Phosphorylated FOXO1 induces cell proliferation, inhibits cell apoptosis, and increases cell invasion and metastasis. Moreover, it also promotes angiogenesis and reduces cell apoptosis in chemotherapy and radiotherapy (Dansen and Burgering, 2008; Zhang B. et al., 2015). AKT phosphorylation was induced by phospho-FOXO1 in NSCLC (Maekawa et al., 2009). However, the involvement of FOXO1 in inhibiting NSCLC cell proliferation is unrevealed.

PI3K/Akt/mTOR signaling pathway has been well characterized and recognized to play essential roles in lung cancer cell proliferation and survival. c-JUN was frequently overexpressed in NSCLC cells. miR-3188 was reported to suppress nasopharyngeal carcinoma cell growth through FOXO1-mediated mTOR-p-PI3K/AKT-c-JUN pathway. In this study, we investigated the interaction of miR-3188, mTOR, and FOXO1 in NSCLC cells. We hypothesized that miR-3188 also inhibit NSCLC cell proliferation via the same mTOR-p-PI3K/AKT-c-JUN signaling pathway. Indeed, we found miR-3188 expression was significantly higher in BEAS-2B cells than in NSCLC cells. miR-3188 mimics inhibited NSCLC cell growth both *in vitro* and *in vivo*. Overexpression of miR-3188 downregulated protein expression of mTOR and p-mTOR in NSCLC cells. And mTOR overexpression reverses inhibition of cell proliferation by miR-3188. More importantly, miR-3188 coordinates with FOXO1 through PI3K/AKT/c-JUN pathway. As such, miR-3188 may negatively modulate NSCLC cell growth by a FOXO1-modulating mTOR-p-PI3K/AKT-c-JUN signaling pathway. Our results suggested that miR3188 might be a potential therapeutic target for NSCLC treatment.

## Materials and Methods

### Cell Culture and Synchronization

Two NSCLC cell lines (A549 and H1299) and a human lung epithelial BEAS-2B cell line were obtained from Shanghai Cell Bank of the Chinese Academy of Sciences. NSCLC cell lines were cultured in RPMI-1640 (Invitrogen, Carlsbad, United States) supplemented with 10% fetal calf serum (FCS; Hyclone, Invitrogen, Carlsbad, United States). BEAS-2B was cultured in defined Keratinocyte serum free medium (KSFM, Invitrogen Carlsbad, United States) supplemented with epidermal growth factor (Invitrogen, Carlsbad, United States). The cells were maintained at 37°C with 10% CO_2_ in a humidified atmosphere.

In order to synchronize cells into G0 phase, NSCLC cells were starved with 0.1% FCS RPMI-1640 for 24–48 h. Cells were then further starved in serum free medium for another 48 h.

### Cell Transfection

FOXO1, c-JUN and mTOR siRNA or miR-3188 mimics and related inhibitor were obtained from RiboBio Inc. (Guangzhou, China). The sequences of primers used for miR-3188 mimics were: Sense 5′AGAGGCUUUGUGCGGAUACGGGG3′, Antisense 3′UCUCCGAAACACGCCUAUGCCCC5′. The sequences used for miR-3188 inhibitor was: 5′CCCCGUAUCCGCACAAAGCCUCU3′. mTOR and c-JUN plasmids were obtained from Biosense Technologies (Guangzhou, China). PI3K inhibitor Ly294002 was purchased from Sigma (St. Louis, United States). NSCLC cells were seeded onto a 6- or 96-well plate at 30–50% confluence before indicated transfection. Cells were transfected with plasmid, siRNA or miRNAs by using TurboFect siRNA Transfection Reagent (Fermentas, Vilnius, Lithuania) following the manufacturer’s protocol. Cells were harvested 48–72 h later for further experiments.

### Western Blotting

Cells were seeded into a 6-well plate and harvested when they reached 90–100% confluence. The detailed method was based on a previous publication (Zhang L. et al., 2015). Antibodies included anti-FOXO1 (2880, 1:1000, CST), mTOR (04-385, 1:1000, millipore), p-mTOR (2971, 1:1000, CST), CCND1 (ab134175, 1:1000, Abcam), p21 (ab109199, 1:1000, Abcam), c-JUN (9165, 1:500, CST), AKT (9272, 1:1000, CST), pAKT (Ser473, 9271, 1:1000, CST), PI3K (4292, 1:1000, CST), p-PI3K (Tyr458, 4228, 1:1000, CST), p27 (2552, 1:1000, CST), and β-actin (ab8227, 1:1000, Abcam). The signal was visualized by using the Western Lightning^®^ ECL Pro Enhanced Chemiluminescence Substrate (PerkinElmer, United States) and exposed to X-ray film (Fujifilm, Japan).

### Colony Formation Assay

For colony formation assay, 100 cells/well NSCLC cells were cultured in 6-well culture plates. Cells were incubated at 37°C for 2 weeks. Colonies were stained with hematoxylin solution after 2 times washing with PBS. Cell clusters include more than 50 cells were identified as colonies. Colony counting was performed using a microscope (IX83; Olympus).

### EdU Incorporation Assay

EdU incorporation assay was performed as previously described (da Silva et al., 2013). Proliferating NSCLC cells were determined through the Cell-Light EdU Apollo 488 or 567 *in vitro* Imaging Kit (RiboBio, Guangzhou, China) based on the manufacturer’s instruction. Briefly, NSCLC cells were incubated with 10 mM EdU for 2 h and then fixed with 4% paraformaldehyde. The fixed cells were permeabilized in 0.3% Triton X-100 for 5 min and stained with dyes accordingly. Cell nuclei were stained with DAPI (5 mg/ml) for 10 min. The number of EdU-positive cells was observed using a fluorescent Nikon microscope.

### *In vivo* Tumorigenesis in Nude Mice

Mice (BALB/C, nu/nu, 6–weeks-old, female) were subcutaneously injected with A549 and H1299 cells (5 × 10^6^/100 μl) transfected with miR-3188, FOXO1 or the control (*n* = 5 per group). All mice were housed in a pathogen-free conditions with controlled temperature (21 ± 2°C) and 12 h light-dark cycle. Food and water were available freely for mice. Three weeks later, the mice were sacrificed and tumor tissues collected for immnunohistochemical analysis.

### Immunohistochemical Staining

Paraffin sections obtained from animal model were used for immunohistochemical staining for FOXO1, mTOR, Ki67, PCNA, or c-JUN expression. The results of immunohistochemical staining tissue sections were evaluated by two pathologists independently. The antibodies used were as follows: rabbit anti-FOXO1 (ab39670, 1:200, Abcam), anti-mTOR (04-385, 1:200, millipore), anti-PCNA (10205-2-AP, 1:50, PTG), anti-Ki67 (Ab16667, 1:50, Abcam), and anti-JUN (24909-1-AP, 1:200, PTG).

### Statistical Analysis

Statistical analysis was performed using SPSS 21.0 software. Data are presented as mean ± SD. Student’s *t*-test was used to compare between two groups and one-way ANOVA (analysis of variance) analysis for multiple groups. All statistical tests were two-sided, and single, double and triple asterisks indicate statistical significance, ^∗^*P* < 0.05, ^∗∗^*P* < 0.01, and ^∗∗∗^*P* < 0.001.

## Results

### miR-3188 Suppresses NSCLC Cell Growth

**FIGURE 1:**
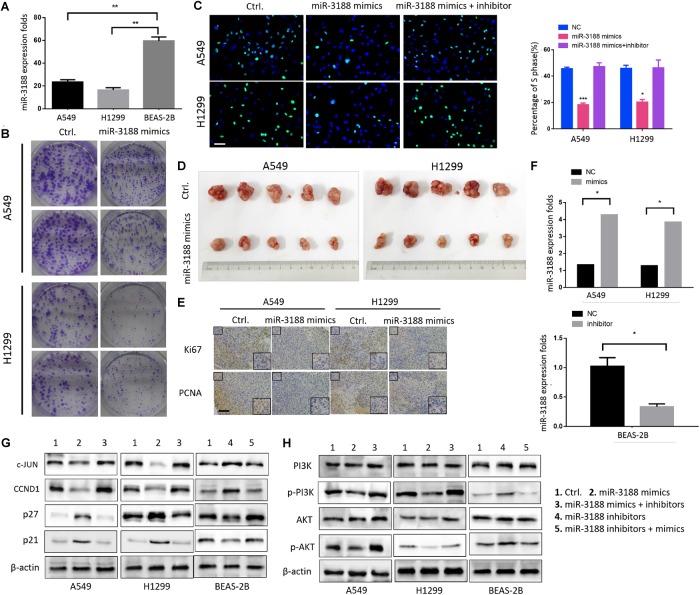
miR-3188 inhibits NSCLC cells proliferation through PI3K/AKT inactivation. **(A)** RT-PCR on miR-3188 expression in NSCLC cells and normal lung epithelial BEAS-2B cells. Mean ± SD, ^∗∗^*P* < 0.01. **(B)** Colony formation, and **(C)** EdU incorporation assays on NSCLC cells performed after transfection with control (ctrl.), miR-3188 mimics, or inhibitor as indicated. Scale bar: 15 μm. **(D)** Tumorigenesis of NSCLC cells before and after transfection with miR-3188 mimics; *n* = 5/group. **(E)** Representative Ki67 and PCNA immunohistochemical staining of A549 and H1299 primary cancer tissues. Scale bar: 20 μm. **(F)** RT-PCR on relative mRNA expression of miR-3188. Student’s *t*-test, mean ± SD, ^∗^*P* < 0.05. **(G,H)** Western blotting on expression of PI3K, AKT, p-PI3K, p-AKT, c-JUN, CCND1, p27 and p21 following transfection of miR-3188 inhibitor or mimics in A549, H1299, and BEAS-2B cells. β-actin was used as a loading control.

To investigate the involvement of miR-3188 in NSCLC development, we first detected its expression in human lung epithelial BEAS-2B cells and NSCLC A549 and H1299 cells. It was found that miR-3188 is overexpressed in NSCLC cells but its expression is low in BEAS-2B cells (Figure 1A). To further identify its biological function in NSCLC, miR-3188 mimics or inhibitors were, respectively introduced into A549 and H1299, or BEAS-2B cells. More than twofold increase in miR-3188 expression was observed in A549 and H1299 cells treated with miR-3188 mimics compared with the control group by qRT-PCR analysis (Student’s *t*-test, with *P* < 0.05 for both, Figure 1F). To examine the effect of miR-3188 expression on NSCLC cell growth *in vitro*, colony formation and Edu incorporation assay were performed. As expected, both A549 and H1299 cells transfected with miR-3188 mimics formed significantly less colonies (Figure 1B). Furthermore, the result of Edu incorporation assay shows that miR-3188 mimics efficiently decreased the number of cells in S phase, and this reduction was rescued by treating cells with miR-3188 inhibitor (Figure 1C). These results indicate that miR-3188 is capable of inhibiting cell proliferation and inducing G1/S transition in NSCLC cells.

Next, an *in vivo* tumor formation experiment was performed through subcutaneous injection of A549-miR-3188 and H1299-miR-3188 or control cells into nude mice. Three weeks later, it was found that tumor size of the mice injected with A549-miR-3188 and H1299-miR-3188 cells was smaller than that of control cells (Figure 1D). Moreover, Ki67 and proliferating cell nuclear antigen (PCNA) expression were also lower in tumor tissues compared to that of control group (Figure 1E). These results suggest that miR-3188 inhibit tumorigenesis both *in vitro* and *in vivo*.

To investigate the mechanisms on how miR-3188 inhibits NSCLC cell proliferation, we performed Western blotting analysis on several biomarkers that regulate cell proliferation. It was found that miR-3188 overexpression reduced c-JUN and CCND1 expression, but increased expression of p27 and p21. miR-3188 inhibitors reverse the expression of these proteins (Figure 1G). Furthermore, p-PI3K and p-AKT expression reduced in miR-3188-overexpressing NSCLC cells (Figure 1H). These results indicate that phosphoinositide-3-kinase (PI3K)/AKT inactivation and c-JUN downregulation might be the mechanisms by which miR-3188 suppresses cell growth.

### miR-3188 Targets mTOR

**FIGURE 2:**
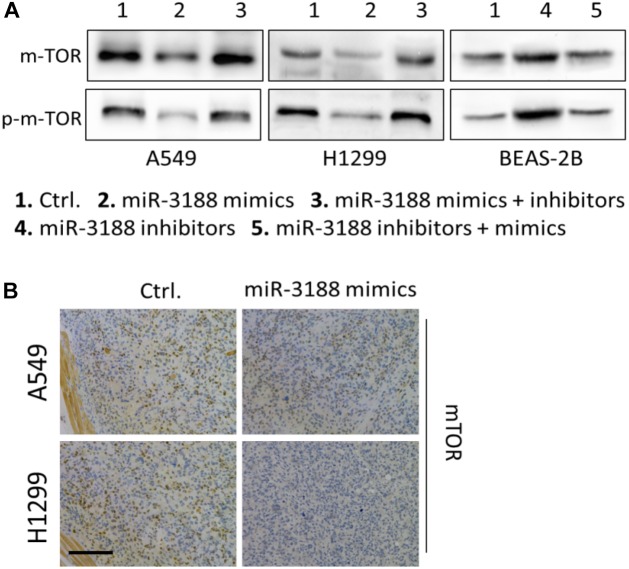
miR-3188 regulates mTOR expression. **(A)** Western blotting on expression of mTOR and p-mTOR in miR-3188-overexpressing or miR-3188-inhibited A549, H1299 and BEAS-2B cells. β-actin was used as a loading control. **(B)** immunohistochemistry on mTOR expression in tumor tissue derived from A549 and H1299 cells. Scale bar: 20 μm.

It has been reported that mTOR is a direct target of miR-3188 (Zhao et al., 2016). We found that overexpression of miR-3188 downregulated protein expression of mTOR and p-mTOR in NSCLC cells. In contrast, miR-3188 downregulation decreased mTOR and p-mTOR levels in BEAS-2B cells (Figure 2A). In mouse xenografts, immunohistochemistry in tumors from A549- and H1299-miR-3188 cells showed that mTOR expression was significantly downregulated (Figure 2B). Collectively, these data suggest that miR-3188 might directly target mTOR and reduce its expression, leading to inhibition of proliferation and tumorigenesis of NSCLC cells.

### mTOR Overexpression Reverses Inhibition of Cell Proliferation by miR-3188

**FIGURE 3:**
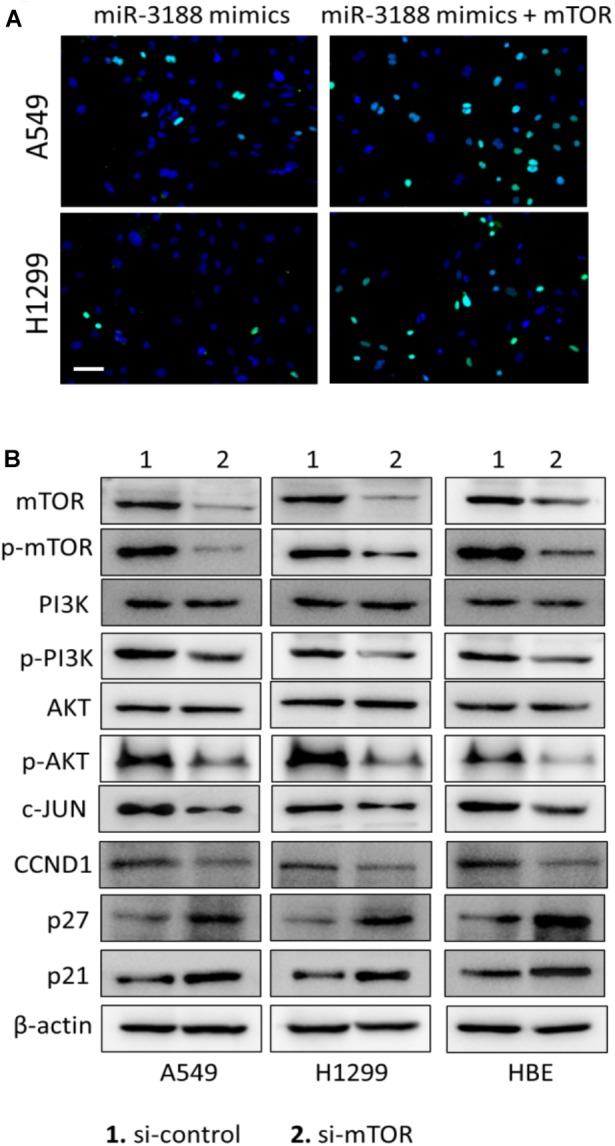
mTOR expression restores miR-3188-mediated inhibition of cell proliferation. **(A)** EdU incorporation assay on cells transfected with control, ectopic mTOR or miR-3188 as indicated. Scale bar: 15 μm. **(B)** Western blotting on mTOR, p-mTOR, PI3K, P-PI3K, AKT, P-AKT, CCND1 and c-JUN expression in A549, H1299 and BEAS-2B cells. β-actin served as a loading control.

EdU incorporation assay indicated that mTOR overexpression in transfected miR-3188-overexpressing NSCLC cells resulted in increasing cell growth (Figure 3A). In mTOR overexpression groups, more cells in phase S were observed in both A549- and H1299-miR-3188 cells, suggesting that mTOR overexpression can restore NSCLC cell proliferation which was inhibited by miR-3188. Also, we observed that mTOR knockdown by siRNA remarkably reduced the expression of mTOR, p-mTOR, p-PI3K, p-AKT, CCND1 and c-JUN, and enhanced expression of p27 and p21 (Figure 3B). These results further confirmed that miR-3188 suppresses cell growth by directly targeting mTOR.

### FOXO1 Suppresses Cell Growth

**FIGURE 4:**
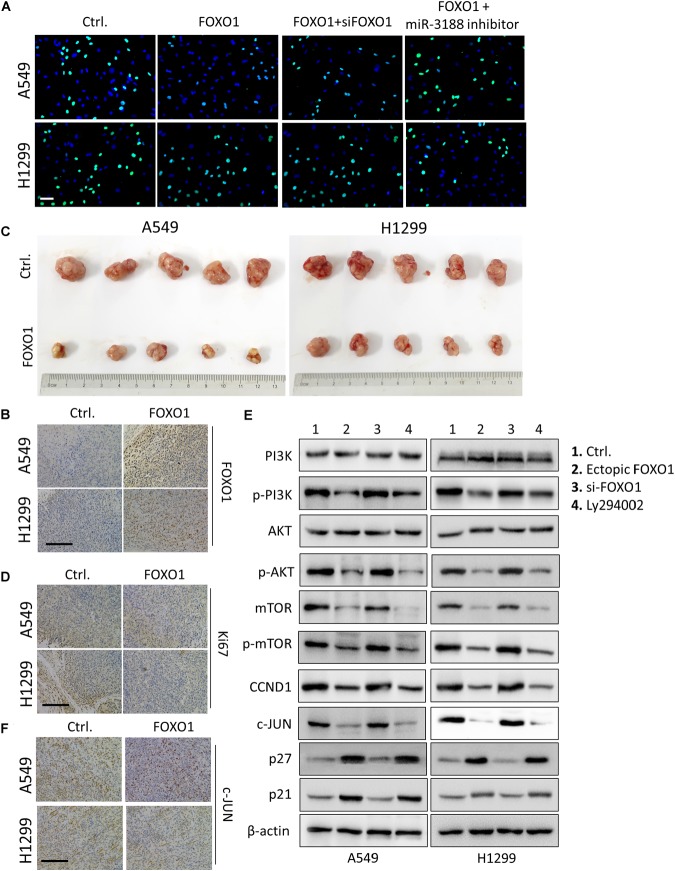
FOXO1 inhibits NSCLC cells proliferation through PI3K/AKT pathway. **(A)** EdU incorporation assay on A549 and H1299 cells transfected with mock and/or FOXO1, siRNA as indicated. Scale bar: 15 μm. **(B)** Immunohistochemical staining of FOXO1 in xenograft tumors. Scale bar: 15 μm. **(C)** Reduced tumorigenicity in FOXO1 overexpressing A549 and H1299 cells; *n* = 5/group. **(D)** Immunohistochemistry of Ki67 in xenograft tumors. Scale bar: 20 μm. **(E)** Western blotting on PI3K, p-PI3K, AKT, p-AKT mTOR, p-mTOR, c-JUN, CCND1, p27 and p21 expression in NSCLC cells transfected with control and FOXO1, siFOXO1 or Ly294002 as indicated. β-actin served as a loading control. **(F)** Immunohistochemical staining of c-JUN in FOXO1 overexpressing xenograft tumors. Scale bar: 20 μm.

It has been reported that FOXO1 induced PI3K/AKT signaling in gastric cancer. To evaluate the role of FOXO1 on NSCLC cell proliferation, we transfected a FOXO1 overexpressed lentiviral vector into NSCLC cells. FOXO1 markedly inhibited cell-cycle G1/S transition in NSCLC cells documented by EdU incorporation assay (Figure 4A). To further confirm that FOXO1 suppress NSCLC cell growth, *in vivo* tumorigenesis experiment was performed in nude mice. Successful overexpression of FOXO1 was confirmed by immunohistochemistry in A549 and H1299 tumors (Figure 4B). Tumor volumes were significantly smaller in tumors of FOXO1-overexpressing A549 and H1299 cells compared to control cells (Figure 4C). Ki67 expression in these tumors was also lower than that in controls (Figure 4D). These results indicate that FOXO1 negatively regulates tumor growth *in vivo*.

FOXO1 overexpression reduced the expression of p-PI3K, p-AKT, mTOR and p-mTOR. Moreover, we observed that overexpression of FOXO1 also downregulated expression of c-JUN and CCND1 and upregulated expression of p21 and p27. Indeed, FOXO1 knockdown by siRNA exhibited the opposite results as shown in Figure 4E. Additionally, the effect of FOXO1 on the expression of p-PI3K, p-AKT, mTOR, p-mTOR, c-JUN, CCND1, p21 and p27 was significantly reduced in cells treated with Ly294002, the specific PI3K inhibitor (Figure 4E).

It has been reported that miR-3188 expression was downregulated by c-JUN, which is a downstream regulator of the PI3K/AKT pathway. Immunohistochemical staining result in FOXO1-overexpressing tumor tissues of mice showed decreased expression of c-JUN (Figure 4F). These results indicate that FOXO1 may mediate NSCLC cell growth and cell-cycle progression through the PI3K/AKT/c-JUN signaling pathway.

### miR-3188 Coordinates With FOXO1 Through PI3K/AKT/c-JUN Pathway

**FIGURE 5:**
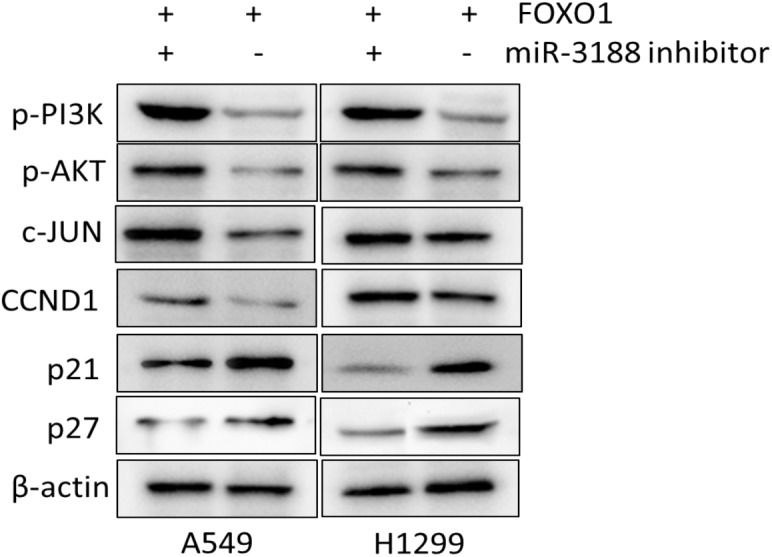
FOXO1 and miR-3188 modulates expression of signaling molecules in A549 and H1299 cells. Western blotting on expression of p-PI3K, p-AKT, c-JUN, CCND1, p21 and p27 in FOXO1 overexpression and miR-3188 inhibition cells. β-actin served as a loading control.

**FIGURE 6:**
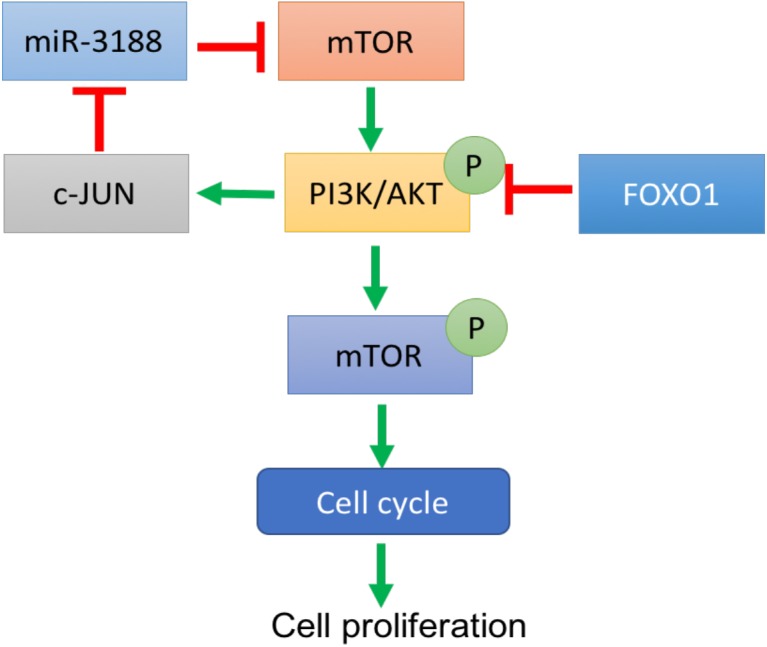
A schematic diagram of FOXO1-mediated miR-3188-mTOR-p-PI3K/AKT-c-Jun signaling feedback loop in NSCLC.

A previous study showed that miR-3188 is a positive modulator of FOXO1 (Zhao et al., 2016). miR-3188 inhibitor significantly rescue cell proliferation in Edu assay (Figure 4A). Expression of p-PI3K, p-AKT, c-JUN and CCND1 was enhanced, while expression of p27 and p21 were decreased in FOXO1-overexpressing NSCLC cells with miR-3188 inhibitor treatment, (Figure 5). These results indicate that miR-3188 coordinates with FOXO1 in suppressing NSCLC cell growth and support that the interaction of miR-3188 and FOXO1is through PI3K/AKT/c-JUN signaling pathway (Figure 6).

## Discussion

miRNAs have been reported to be associated with various types of cancers (Liu and Gao, 2016; Zheng et al., 2017). However, the role of miR-3188 in NSCLC development has not been reported. In this study, we demonstrated that miR-3188 significantly suppressed proliferation and G1/S cell-cycle transition in NSCLC cells as well as growth of tumor xenografts, indicating that miR-3188 might be a tumor suppressor in NSCLC.

It is well known that tumor cell proliferation is associated with cell-cycle progression (Collins et al., 1997). We found that miR-3188 regulate NSCLC tumorigenesis by affecting cell cycle. We also identified that the role of miR-3188 is associated with other key signaling molecules including mTOR, PI3K/AKT and c-JUN, that suppress cell cycle transition, thus inhibiting cell growth.

mTOR is an oncogene and constitutively activated PI3K/Akt signaling pathway was observed in almost every types of tumors (Populo et al., 2012; Xie et al., 2016; Wang et al., 2017) including NSCLC (Gridelli et al., 2008). It is well known that PI3K/Akt is involved in cell survival, growth, proliferation, repair, migration and angiogenesis (Lucas et al., 2010; Liu et al., 2014; Henderson et al., 2015). In cancer cells, activation of signaling pathway could be realized by gene mutation or upstream signaling molecules (Porta et al., 2014; Lien et al., 2016). It has been well documented that mTOR could form a negative feedback loop with PI3K/AKT (Efeyan and Sabatini, 2010; Rozengurt et al., 2014; Carneiro et al., 2015). In this study, we found that mTOR is one of the direct targets of miR-3188 and PI3K/AKT signaling was inhibited by miR-3188 overexpression. However, PI3K/AKT signaling activation was inhibited by mTOR in breast cancer (Khan et al., 2013; Paplomata and O’Regan, 2014), indicating differential effect of mTOR in different type of cancers. As such, c-JUN and p-mTOR that are PI3K/AKT downstream molecules increased in mTOR knockdown NSCLC cells, that was consistent with in miR-3188 overexpression cells. Furthermore, mTOR overexpression enhanced NSCLC cell proliferation suppressed by miR-3188. These results confirm that miR-3188 inhibits PI3K/AKT pathway by suppressing mTOR, leading to downregulation of c-JUN and p-mTOR.

c-JUN has been well known as a key player of cell proliferation (Shaulian and Karin, 2001), invasiveness and metastasis (Peng et al., 2016), and is one of the PI3K/AKT pathway downstream components (Cai et al., 2010; Zhang et al., 2014). Subsequent experiments confirmed that c-JUN negatively mediates miR-3188 expression indicating the formation of a complex miR-3188-mTOR–pPI3K/AKT-c-JUN loop in NSCLC.

It was reported that FOXO1 is a negative regulator in various types of cancers (Zhang et al., 2012; Hou et al., 2016). Consistent with previous reports, NSCLC cells in G1/S cell cycle transition and cell growth was significantly suppressed by FOXO1. In this study, FOXO1 overexpression inhibited PI3K/AKT signaling pathway mediated cell-cycle process in NSCLC cells. However, this finding is not consistent with that in gastric cancer (Park et al., 2014). In NSCLC cells, c-JUN, p-mTOR and total mTOR expression were downregulated. Similar to miR-3188, FOXO1 also inhibits NSCLC cell proliferation through suppressing PI3K/AKT mediated cell-cycle process.

We also found that miR-3188 failed to promote the expression of FOXO1, indicating that miR-3188 might be a downstream molecule of FOXO1. Through blocking mTOR-mediated p-PI3K/AKT/p-mTOR signaling activation, effect of miR-3188 was suppressed. In this case, FOXO1 lost its inhibitory effects on c-JUN and cell cycle and subsequently induced cell growth in NSCLC cells. Taken together, miR-3188 could be a downstream component of FOXO1 signaling.

In summary, our study supports that miR-3188 could form a negative feedback loop through mTOR/PI3K/AKT/c-JUN pathway. This pathway was regulated by FOXO1 which results in suppression of cell proliferation in NSCLC cells.

## Author Contributions

TL and CW conceived the project. CW, EL, WL, and JC performed experiments. CW, EL, WL, JC, and TL analyzed the results. TL and EL wrote the first draft. All authors revised the manuscript. TL edited and approved the manuscript.

## Conflict of Interest Statement

The authors declare that the research was conducted in the absence of any commercial or financial relationships that could be construed as a potential conflict of interest.
